# A Dynamic Navigation Model for Unmanned Aircraft Systems and an Application to Autonomous Front-On Environmental Sensing and Photography Using Low-Cost Sensor Systems

**DOI:** 10.3390/s150921537

**Published:** 2015-08-28

**Authors:** Andrew James Cooper, Chelsea Anne Redman, David Mark Stoneham, Luis Felipe Gonzalez, Victor Kwesi Etse

**Affiliations:** Queensland University of Technology, Australian Research Centre for Aerospace Automation (ARCAA), 2 George St, Brisbane QLD 4000, Australia; E-Mails: andy90@me.com (A.J.C.); chelsea.redman@connect.qut.edu.au (C.A.R.); david.stoneham@connect.qut.edu.au (D.M.S.); victorkwesi.etse@connect.qut.edu.au (V.K.E.)

**Keywords:** unmanned aircraft system, predictive path planning, societal integration, dynamic waypoint navigation, aerial photography, aerial filming

## Abstract

This paper presents an unmanned aircraft system (UAS) that uses a probabilistic model for autonomous front-on environmental sensing or photography of a target. The system is based on low-cost and readily-available sensor systems in dynamic environments and with the general intent of improving the capabilities of dynamic waypoint-based navigation systems for a low-cost UAS. The behavioural dynamics of target movement for the design of a Kalman filter and Markov model-based prediction algorithm are included. Geometrical concepts and the Haversine formula are applied to the maximum likelihood case in order to make a prediction regarding a future state of a target, thus delivering a new waypoint for autonomous navigation. The results of the application to aerial filming with low-cost UAS are presented, achieving the desired goal of maintained front-on perspective without significant constraint to the route or pace of target movement.

## 1. Introduction

Dynamic waypoint navigation capabilities of unmanned aircraft systems (UAS) are considered an important component of UAS use in environmental sensing [1]. This is especially true in operating environments where the obstacles or objects of interest exhibit non-deterministic dynamics [2]. These characteristics are more common for real-world objects than the static types that can be handled using *a priori* information and static waypoint navigation capabilities [1]. As the UAS progress from laboratories and “test” environments, dynamic navigation capabilities will become essential to maintaining safety requirements.

Autonomous collision avoidance of either ground-based obstacles or other airspace users is a dynamic navigation technology that has been extensively researched throughout the past decade across a broad range of applications [1,3,4,5]. This research has been further developed and applied extensively across the spectrum of low-cost ground-based [6] systems through to applications for general aviation [7]. Many of these avoidance systems involve *a priori* known “targets” or deterministic “targets”, where their location can be calculated throughout the process, and a number of statistical-based solutions to this class of problem exists. This technology has resulted in significant advancement in the area of UAS navigation [8]. The resulting safety improvement has also had a positive impact on public perception of UAS operations [9].

UAS autonomous following is another area of research that has been explored [10,11,12,13,14,15] and is different from the previously-discussed class of problem in that the “target” is non-deterministic, and as such, no solution exists for the calculation of its location at a given future time. Rafi *et al.* [10] discuss a new algorithm for the autonomous navigation of fixed wing unmanned aerial vehicles (UAVs) following a moving target. The UAV has physical constraints on airspeed and manoeuvrability, whilst the target is not considered to be constrained. The authors use a single circular pattern navigation algorithm that works for targets moving at any speed and in any pattern, whereas other methods switch between different navigation strategies in different scenarios. Additionally, the system takes into consideration that the aircraft also needs to visually track the target using a mounted camera, which is controlled by the algorithm according to the position and orientation of the aircraft and the position of the target.

Similarly, Zengin and Dogan [13] present a rule-based intelligent guidance strategy for the autonomous pursuit of mobile targets by UAVs in areas that contain threats, obstacles and restricted regions, while Wise and Rysdyk [12] discuss different methodologies for coordinating multiple UAVs for autonomous target tracking to further improve performance and stability. Zengin and Dogan [13] used a probabilistic threat exposure map (PTEM) as the mathematical formulation of the area of operation for the guidance strategy to make intelligent decisions based on a set of defined rules. The authors also used a least-squares estimation and kinematic relations to estimate target states based on noisy measurements. Wise and Rysdyk [12] also discuss some of the probable limitations, like wind, and how this limitations impact on single UAV autonomous tracking methodologies.

Considering a number of related factors, Sujit *et al.* [11] present a detailed summary and evaluation of UAV path following algorithms. In this work, the authors describe and compare five path following algorithms and guidance laws that are robust to disturbances using total cross-track error and control effort performance metrics. Clearly, autonomous following has seen significant research and development, including the application of a broad range of methodologies, models and analysis techniques to a broad range of target types, behaviours and environments.

This class of problem has commercial utility in the area of sports coverage and film production [10], which was evidenced by the use of remotely-piloted aircraft systems (RPAS) for sports coverage at the Sochi 2014 Winter Olympics. Increased public exposure has contributed to an increased demand for RPAS filming solutions, with Silicon Valley proposing several purpose-built UAS featuring autonomous following capabilities. These projects have been crowd funded for more than US$1,500,000.00 to date [16,17], confirming an interest in this capability. Of note is that many of these proposed UAS have significant limitations, such as a lack of collision avoidance capability (static or deterministic targets) and an inability to effectively track to a position in front of a target’s heading during unconstrained navigation.

Even though related works have considered the application of controlled navigation in other systems, the reallocation of waypoints in higher level user-controlled navigation specifically for UAS is a different, albeit similar area of interest. Dynamic path planning can generally be separated into two levels: one where the UAS has the ability to perform trajectory planning to move from one given location to another, where the locations are provided dynamically [18], and the second is a higher level, where, in addition to trajectory planning, the UAS is able to assign waypoints in response to an observed cue. The area of research addressed in this paper is based on the second level, where we address the problem of assigning waypoints based on a hypothesis of the “target” state.

Freedom from pre-allocated waypoint navigation could be achieved for low-cost UAS through the development of a navigation system that features autonomous and dynamic waypoint assignment capabilities in response to real-time “target” position data acquisition and processing. This paper will focus on the application of this capability to a filming UAS, whose objective is to film an athlete (or similar “target”) from an autonomously-maintained front-on perspective.

With the increasing popularity of low-cost UAS, there is also a need to develop capabilities that encourage safe operation [19]; however, in some cases, increased capabilities can also help to mitigate the risk from other activities. For example, there is significant risk involved in filming action sports. Often, camera equipment is operated in one of three configurations; several strategically-placed static camera operators, a camera operator in a manned helicopter or a camera operator riding in reverse orientation on a motorcycle. These configurations often involve camera operators situating themselves in precarious positions in an effort to attain the desired perspective, which is clearly undesirable in terms of operator and public safety. Development of UAS with increased capabilities would help to mitigate some of this risk.

In an effort to stop inexperienced users manually controlling UAS, a robust autonomous system [20] is required to limit user control to some degree. Conventional UAS filming techniques require that either a UAS is manually piloted or that the “target” adheres to a predefined route and pace. Dynamic waypoint assignment and autonomous navigation capabilities could remove this requirement whilst still achieving the desired creative outcome.

In addition to developing this model for dynamic waypoint navigation, a mobile phone-based graphical user interface (GUI) was developed. This interface provides a user with some basic operational control, including access to take-off and land commands and altitude and proximity settings; however, the remainder of control was handled by the on-board autopilot system. Note that there is no collision avoidance in this work; similar to current operations in which a manned aircraft or helicopters fly at sufficient altitude and in areas clear of obstacles. Note also that we rely fully on Global Position System (GPS) information, with less than 10-m resolution accuracy being enough for the algorithm to be functional. Additionally, the system is adaptable to function on any set of two-dimensional positional data.

## 2. Prediction Methods and Algorithm

**Figure 1:**
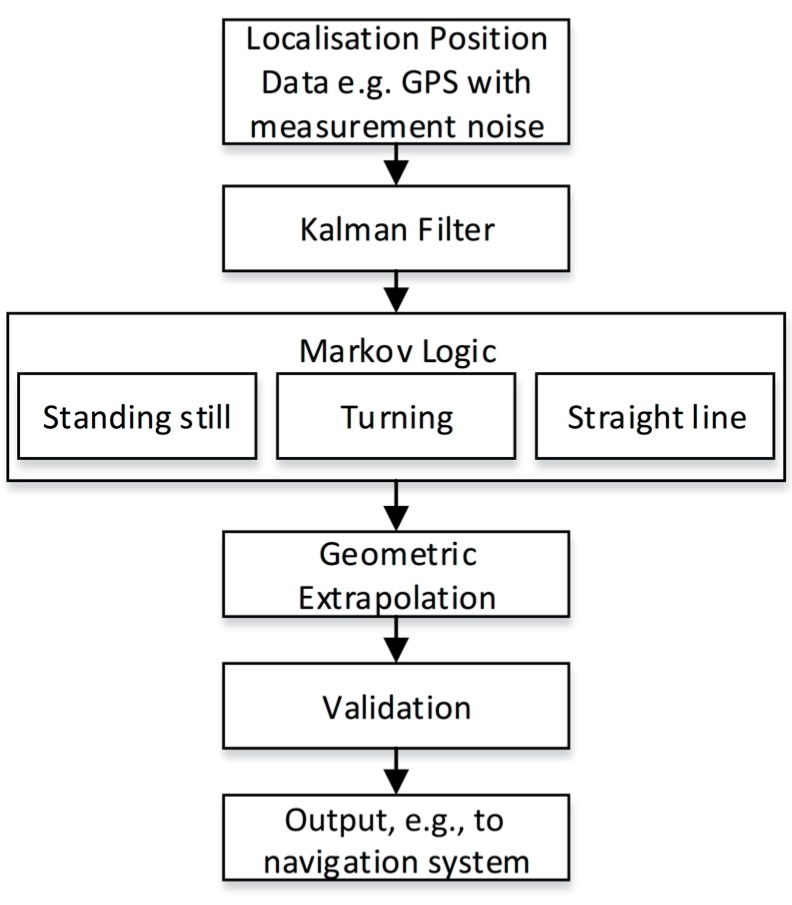
Block algorithm of the prediction model.

The prediction algorithm developed consisted of six major blocks to process time varying data in a real-time environment, as seen in Figure 1.

A low-cost mobile device provides positional data (GPS) about the target user (e.g., a sportsperson) as the input to the algorithm. Measurement noise is part of the location coordinates, and a Kalman filter is used to give a better representation of human movement and true position. Markov logic is then applied using previous data points to determine the navigational behaviour of the user at each time interval. During initialisation, a number of assumptions are made in order to populate state transition and emission tables; however, as data are collected, the algorithm is able to update these based on the distributions of newly-sampled data. The algorithm was designed to recognise some of the major states of a user’s movement at any given time. These states were defined as stationary ${\text{Ω}}_{0}$, linear navigation ${\text{Ω}}_{1}$ and non-linear navigation ${\text{Ω}}_{2}$. Depending on the maximum likelihood case $\hat{s}$, a different algorithm was used to predict a future location taking into account this previous behavioural assumption. Once a state was assigned to the motion of the user, a least-squares regression was implemented for the best fit and to enable extrapolation of the collected points to a possible prediction solution. The Haversine formula was implemented to determine the predicted GPS location based on the subject’s expected relative displacement. The algorithm could then output a predicted GPS coordinate that could be validated to ensure it was within reasonable distance to the user’s previous position. This was implemented by performing a vector magnitude calculation and comparing the result of this to a user-defined maximum travel distance, relative to the most recent target localisation. This is done to ensure that as target velocity increases, predicted positions and resultant UAS displacement does not exceed the range of the telemetry devices being used, and it also helps to enforce sensible predictions. Upon passing this validation, the GPS location is output for external use, for example to a navigation system of an autopilot.

### 2.1. Input Position Data

In the simplest case, GPS receivers utilise GPS satellites to give position estimates [21,22]. In this standalone configuration, a low-cost GPS module was tested and typically exhibited estimates within 10 m of a known target location. It is clear that assisted GPS (AGPS) could be employed to improve this accuracy. This technology typically incorporates GPS data, cell tower trilateration and Wi-Fi network information. Some devices are also able to incorporate inertial measurement unit (IMU) data with AGPS technology in order to yield positional estimate accuracy improvements [21,23]. It should be noted that differential GPS, whilst certainly applicable to this type of work, is not currently available at a price-point in keeping with the desired application of low-cost UAS.

After testing multiple AGPS and IMU + AGPS-enabled mobile devices, the iPhone 5S was found to offer better accuracies in our test location and for our application with an average cross-track error of 2.25 m. This was determined by logging the GPS position output of the smart phone whilst walking between two predefined locations. It should be noted that although the iPhone 5S was chosen for this application, any GPS-enabled device could provide input data, and future developments to this technology could offer significant improvements to the performance of the prediction algorithm.

For simplicity, our application only utilises GPS-based location inputs. Additional inputs could be utilised to increase the performance of the system, such as additional sensors, like Vicon, encoders or image-based localisation. These could be implemented either on the target, the UAS or both the target and UAS and might provide improved accuracy and greater knowledge of the movement characteristics of the target.

### 2.2. Kalman Filter

**Figure 2:**
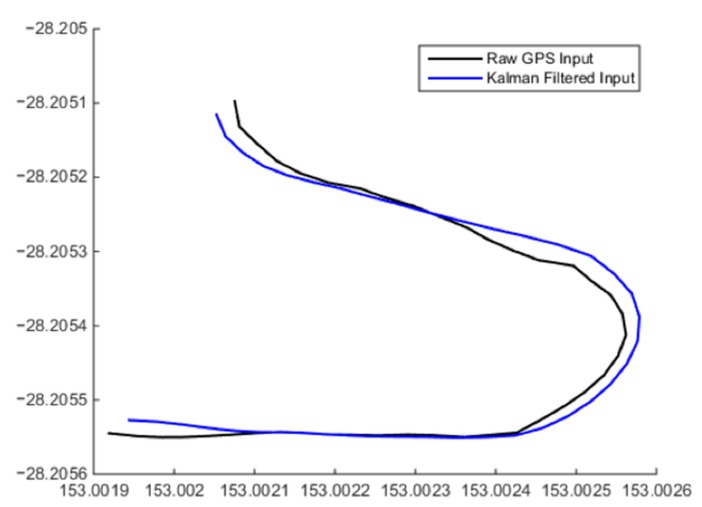
The increased precision of the input GPS coordinates after application of a Kalman filter to produce a more realistic human movement path.

Low-cost GPS receivers generally exhibit a high quantisation error that degrades the accuracy of readings [24]. On the scale of low-cost UAS navigation, the positional accuracy of these systems needs to be as high as possible to allow safe operation of the system. Through the use of a Kalman filter, the precision of these input positional estimates from low-cost GPS receivers can be increased to give a better representation of human movement without significantly increasing the cost of the system, as shown in Figure 2.

The Kalman filter is an efficient mathematical algorithm used for stochastic estimation from noisy sensor measurements by employing both a prediction and observational model to statistically produce noise-corrected state outputs [25]. When applying a Kalman filter to GPS coordinates, the process is simplified slightly, since the location providers output not only a location as longitude and latitude, but also a measure of accuracy (in meters). Therefore, instead of implementing a traditional covariance matrix, the accuracy of the filter can be represented by a single value and an assumed normal distribution.

**Figure 3:**
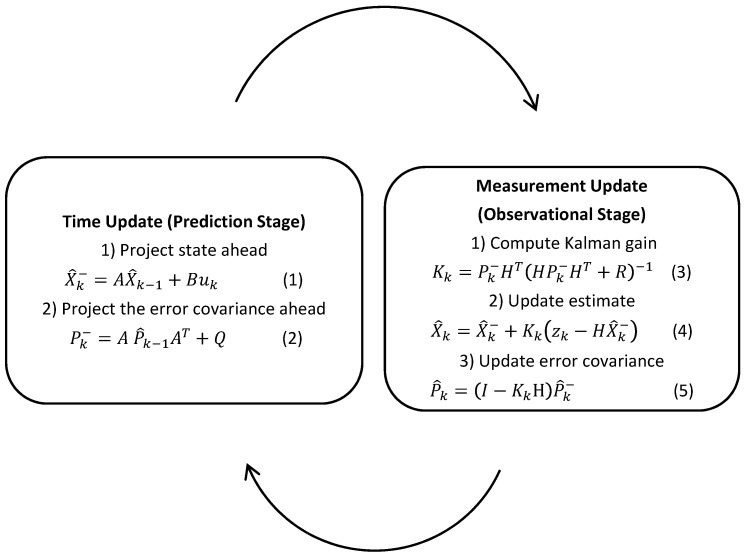
Two-stage approach of Kalman state estimation filtering.

Additionally, many potential targeting systems have a regular or near-regular frequency output of positional data. For example, common low-cost GPS modules might be polled at 50 Hz or some mobile device operating systems call delegate object event handlers with a frequency of approximately 1 Hz. This approximate regularity enables the implementation of iterative filtering over a discrete time domain [26]. The Kalman filter implementation used iterates through two major phases, as shown in Figure 3: the first phase is the prediction state; updating the accuracy of the system using an n evolution prediction model to produce state estimates, (Equation (1)), from a previous state and an instantiation or update of a covariance matrix (Equation (2)) [24,25].

(1) $${\hat{X}}_{k}^{-}=A{\hat{X}}_{k-1}+Bu_{k}$$

(2) $$P_{k}^{-}=A{\hat{P}}_{k-1}A^{T}+Q$$

where:

$P^{-}=\text{prior state covariance matrix}$
A=state transition model
B=control input model
u=control vector
Q=process noise

For the desired application, the location of the target is measured in terms of initial longitude *x*_0_ and initial latitude *y*_0_ prior to operation, and this measurement is input to build the initial state vector. The initial displacement derivatives were assumed zero. The state vector definition and this initial system state are presented in Equation (3). Note that using GPS data in this way approximates the data as orthogonal and makes the algorithm unusable across the antipodal meridian or very close to the poles.

(3) $$X_{k}={\left[\begin{array}{cc} \begin{array}{ccc} x_{k} & y_{k} & {\dot{x}}_{k} \end{array} & \begin{array}{ccc} {\dot{y}}_{k} & {\ddot{x}}_{k} & {\ddot{y}}_{k} \end{array} \end{array}\right]}^{\prime },\;X_{0}=\left[\begin{array}{cc} \begin{array}{ccc} x_{0} & y_{0} & 0\; \end{array} & \begin{array}{ccc} 0\; & \;0\; & 0 \end{array} \end{array}\right]\prime$$

The state transition model is derived simply from Newton’s laws of motion. For simplicity, and since the system should operate in open-air environments and upon a range of targets, which may take different forms, target drag is neglected. A time-discrete state transition model is represented by Equation (4), where *p* is the constant period of measurement, related to the polling or calling frequency. In our testing, this was approximated to one second.

(4) $$A=\left[\begin{array}{cc} \begin{array}{ccc} 1 & 0 & p\\ 0 & 1 & 0\\ 0 & 0 & 1 \end{array} & \begin{array}{ccc} 0 & 0 & 0\\ p & 0 & 0\\ 0 & p & 0 \end{array}\\ \begin{array}{ccc} 0 & 0 & 0\\ 0 & 0 & 0\\ 0 & 0 & 0 \end{array} & \begin{array}{ccc} 1 & 0 & p\\ 0 & 1 & 0\\ 0 & 0 & 1 \end{array} \end{array}\right]$$

The second phase of the filter takes into account the measurement inputs of the system to produce a state estimate by correcting the predicted state and covariance estimates from the first phase of the filter. This is done by first computing the Kalman gain (Equation (3)), then correcting both the state estimates and covariance matrix (Equations (6) and (7)) [24,25].

(5) $$K_{k}=P_{k}^{-}H^{T}{\left(HP_{k}^{-}H^{T}+R\right)}^{-1}$$

(6) $${\hat{X}}_{k}={\hat{X}}_{k}^{-}+K_{k}\left(z_{k}-H{\hat{X}}_{k}^{-}\right)$$

(7) $${\hat{P}}_{k}=\left(I-K_{k}\text{H}\right){\hat{P}}_{k}^{-}$$

where:

$K_{k}=\text{kalman gain}$
H=observation model
R= gaussian white noise covariance
${\hat{X}}_{k}=\text{true state}$
$z_{k}=\text{observation}$
I=identity matrix

This two-stage process is initialised with initial estimates ${\hat{X}}_{k-1}$ and $P_{k-1}^{+}$ and is repeated until filtering ends.

### 2.3. Markov Logic

It is possible that there are known patterns within positional datasets produced by the target (user, sportsperson), and a model that learns from and has an understanding of these patterns may be able to make better use of the input positional data in terms of predicting and outputting a positional estimate. This offers improved prediction accuracies. If these behavioural aspects are apparent in the input data, then the prediction model should account for them; this can be achieved through the use of a hidden Markov model (HMM) [27].

It is assumed that there are at least three categories of navigational traits exhibited by an unassisted human: standing still, moving in a straight line and turning a corner. Note that this assumption does restrict the application scope of the generic prediction model and should be reassessed if applying these predictive navigation methods to non-human targeting, *i.e.*, if a car were to be targeted by the system, an additional “reversing” state could be added. If an assumption is made where the x and y directions are defined as some orthogonally-related compass bearing of position vector s and altitude z is maintained at some constant elevation and therefore disregarded, the categories to be considered are reduced to no change, linear change and non-linear change. These are used to define three states.

State 0 is defined as a state where there is no change in position and the target, e.g., the user, is considered to be stationary.

(8) $$\frac{\Delta x}{\Delta t},\;\frac{\Delta y}{\Delta t}\approx \;0\;\Rightarrow {\text{Ω}}_{0}$$

If there is a linear change in position, the magnitude of the derivative components may change; however, the resultant direction of travel will not. In this state, the target is considered to be moving in a straight line. This is defined as State 1.

(9) $$\left\{x_{t+1}=\;x_{t}\pm a,\;y_{t+1}=\;y_{t}\pm b,\;a:b\;\approx \text{constant}\right\}\;\Rightarrow {\text{Ω}}_{1}$$

where a and b are some scalars derived from the input position coordinates.

Finally, if the constraints of State 0 and State 1 are breached, State 2 defines that the target is turning a corner or otherwise deviating from a linear or stationary sequence of positions.

(10) $$\left\{x_{t+1}=\;x_{t}\pm a,\;y_{t+1}=\;y_{t}\pm b,\;a:b\;\neq \text{constant}\right\}\;\Rightarrow {\text{Ω}}_{2}$$

again, where *a* and *b* are some scalars derived from the input position coordinates.

Depending on the application, the initial state probability matrix can be configured in a number of ways. If the probabilities are known, then the configuration is to match the distribution of the initial state $q_{0}$. If the probabilities are unknown, then they need to be assumed [28]. One of two assumptions are suggested; either $q_{0}={\text{Ω}}_{0}$ or that of an equal distribution of assumed states. Table 1 shows all three states and their associated probability assumptions.

**Table 1:** State matrix probability assumptions.

	$\Omega_{0}$	$\Omega_{1}$	$\Omega_{2}$
$Pr\left(q_{0}\right)\;assumption$	1	0	0
$Pr\left(q_{0}\right)alternative\;assumption$	13	13	13

State transitions and emissions can be configured in much the same way. In the case where transition probabilities ϕ and emission probabilities θ representative of the behavioural characteristics of the target are known, the configuration table is relatively straightforward. In the case that these characteristics are unknown, they must be initially assumed prior to an iterative update. The suggested assumption is an equal distribution of ϕ and θ.

(11) ϕ, θ=[131313131313131313]

A user-defined quantity S of position observations is used to make one T state observation, where each new position observation and the preceding S−1 position observations correspond to a new state observation. Testing of our system at 1 Hz indicated an optimal S value of between four and eight, depending on the target route. In this way, there exists a delay $\frac{S}{f}$, which suggests maximizing the frequency f of position observation reads where possible. Decreasing S may also be possible; however, this would accompany a decrease in sample size and a potential accuracy of geometric state definitions. In the HMM, S−1 observations are used for two separate state observations; S−2 observations are used for three separate state observations, and so on. Furthermore, these observations can be different depending on the characteristics of the positions recorded outside of this reduced set. In this way, the actual current state ${\bar{q}}_{t}$ is never known at the time of reduction, and the emission matrix θ must be used to manage the distribution of probabilities between the predicted ${\bar{q}}_{t}$ and actual $q_{t}$. This adds weight to the assumed initial state, state transition and emission matrices, since they are not updated until S observations are recorded, which suggests that increased position data read frequencies may be preferred.

Upon S observations and by using Equations (12)–(14), the Baum–Welch method was used to iteratively solve the Markov model [29], updating the transition and emission matrices ϕ and θ and also for derivation of a local maximum likelihood $\hat{s}$. The generated dataset may also be able to be used to better populate the initial state, state transition and emission matrices for future tests where the target is expected to exhibit similar navigational behaviour. The calculated local maximum likelihood state case is used to determine which algorithm to apply to the most recent data subset in order to construct a target location prediction.

(12) $$\pi_{i}^{*}=\text{ expected frequency in state }S_{i}\text{ at time }t\;=\;1$$

(13) $$\begin{array}{ll} a_{ij}^{*}= & \text{expected number of transitions from state }S_{i}\text{ to state }S_{j}\\ \; & \;/\text{expected number of transitions from state }S_{i} \end{array}$$

(14) $$\begin{array}{ll} b_{j}^{*}\left(k\right)\;= & \text{expected number of transitions from state}\;S_{j}\;\text{and observing }v_{k}\\ & \;/\text{expected number of transitions from state}\;S_{j} \end{array}$$

### 2.4. GPS Calculations

Taking into account the maximum likelihood navigation characteristics and the GPS-measured velocity vector of the target, an expected future position can be estimated. Spherical trigonometry is used to calculate great circle distances between the GPS coordinates. Approximating the Earth as perfectly spherical simplifies the implementation by enabling Haversine to be applied. It should be noted that this introduces up to a 0.55% error [30]. Assuming typical displacements of less than 100 m, this error can be considered insignificant as compared to the accuracy of the GPS modules in use, as noted previously.

**Figure 4:**
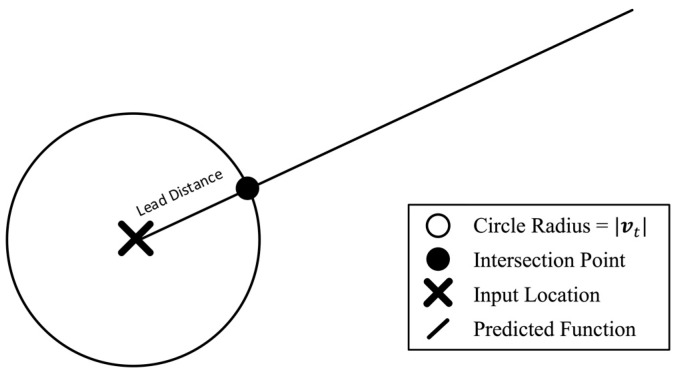
Locating the next GPS point using the intersection of the circle with the predicted function, where the circle has a radius equal to the desired lead distance.

Calculated displacements and associated velocity and acceleration vectors can then be used to geometrically infer prediction coordinates. A circle function with the radius equal to the magnitude of the target velocity $v_{t}$ was centred over the most recent input data position. The intersection of the circle function and the prediction function would then produce the next desired UAS location. As seen in Figure 4, the Haversine formula [31] is applied as follows:

(15) $$a=\sin^{2}\left(\frac{\text{Δφ}}{2}\right)+\cos{\text{φ}}_{1}{\text{*cosφ}}_{2}*\sin^{2}\left(\frac{\text{Δ}\lambda }{2}\right)$$

(16) $$c=2*atan2\left(\sqrt{a},\;\sqrt{\left(1-a\right)}\right)$$

(17) d=Radius of the Earth*c

where:

Δλ is change in longitude
Δφ is change in latitude 
${\text{φ}}_{1}\;is\text{ latitude }1$
${\text{φ}}_{2}\;is\text{ latitude }2$
The radius of Earth was approximated to be 6371 km.

## 3. Simulation Experiments

**Figure 5:**
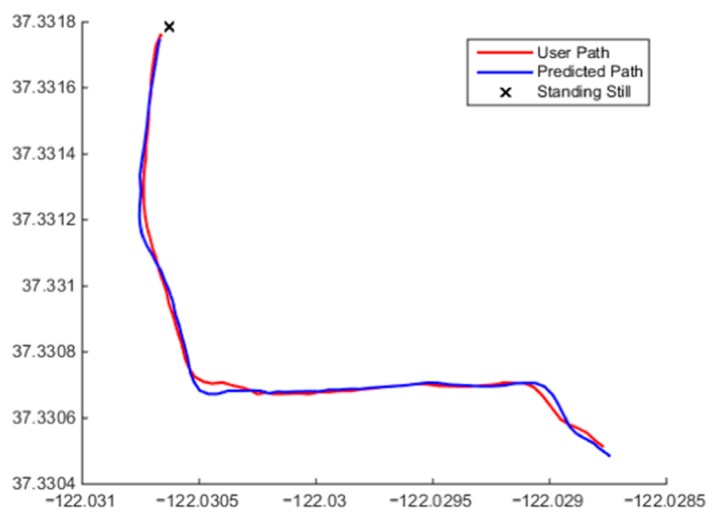
Beginning of the output showing the identification of ${\text{Ω}}_{0}$ followed by switching between predictions methods for states ${\text{Ω}}_{1}$ and ${\text{Ω}}_{2}$ when target movement changes between general linear behaviour to general non-linear behaviour.

**Figure 6:**
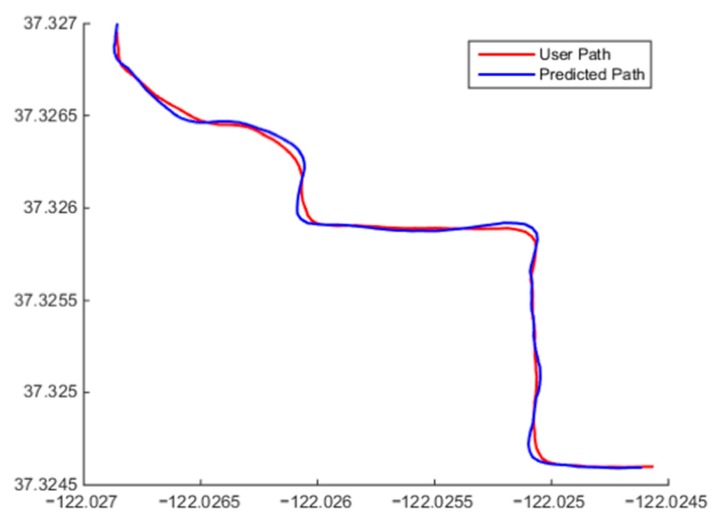
Algorithm predicting ${\text{Ω}}_{2}$ behavior when presented with non-linear target motion and ${\text{Ω}}_{1}$ behavior when presented with approximately linear target motion.

Once the initial model was designed, it was implemented in the MATLAB simulation environment. Data were gathered from the mobile device GPS and other sources and fed into the algorithm to simulate the model response for various datasets. Different routes were taken to ensure adequate testing coverage of the various aspects of the algorithm. Some of the results of these tests can be seen in Figure 5 and Figure 6.

The beginning of a user’s path and the corresponding predicted points are shown in Figure 5. It can be seen that model correctly identified that the user was standing still at the start of the simulation and then began moving in an approximately linear fashion. The algorithm correctly chose ${\text{Ω}}_{0}$ then transitioned to ${\text{Ω}}_{1}$ and produced a linear prediction position sequence. Figure 6 shows a combination of the user continuing in a straight line and exhibiting random non-linear motion. The algorithm fitted both ${\text{Ω}}_{1}$ and ${\text{Ω}}_{2}$ to this dataset. Along the straights, ${\text{Ω}}_{1}$ was fitted and a linear output produced; when the user began turning, ${\text{Ω}}_{2}$ was chosen, and faster reacting polynomial output can be observed. For this simulation, a total average cross-track was determined to be 0.66 m when compared to the user’s path. This means that the predicted points were accurate on average to 0.66 m. It should also be noted that the user did not exhibit a constant speed during testing, and the algorithm was able to recalculate the predicted velocity of the user with every new positional input.

## 4. Flight Test

**Figure 7:**
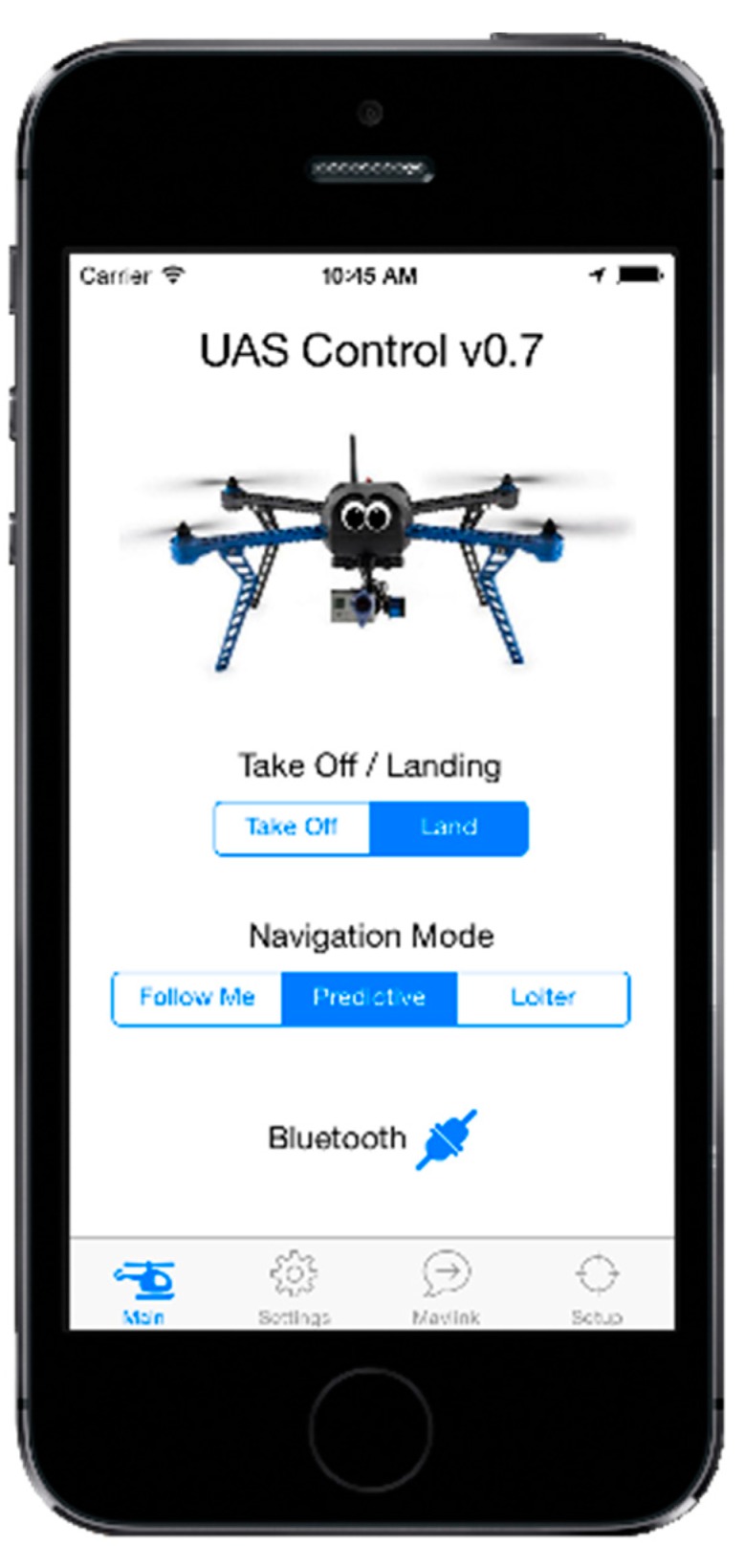
iOS ground control station (GCS) application used for iPhone 5S.

An example application is the integration of the algorithm onto a UAS for the purpose of filming real-time action sports from a front-on perspective. The algorithm was designed to be installed onto a number of platforms and devices, such as a mobile phone, a microprocessor (Arduino), a Raspberry Pi computer or a networked server system. In this case, it was implemented in an iOS application running on an iPhone 5S (Figure 7), which allowed the device to be utilised as the input to the algorithm and to process all data and transmit the desired waypoint coordinates to the UAS over a Bluetooth 4.0 telemetry radio connection. The iOS application was also able to serve as the ground control station (GCS) for the user, providing functional control over the UAS, such as (autonomous) take off, (autonomous) landing, altitude control, predictive mode, loiter mode, follow-me mode and return to launch (RTL), as well as the capability to tune the algorithm variables, such as distance-from-line threshold, stationary threshold and desired lead distance (how far ahead the UAS will be from the user). The user would simply set up these distance parameters, press “take off” and, finally, put the system into predictive mode.

**Figure 8:**
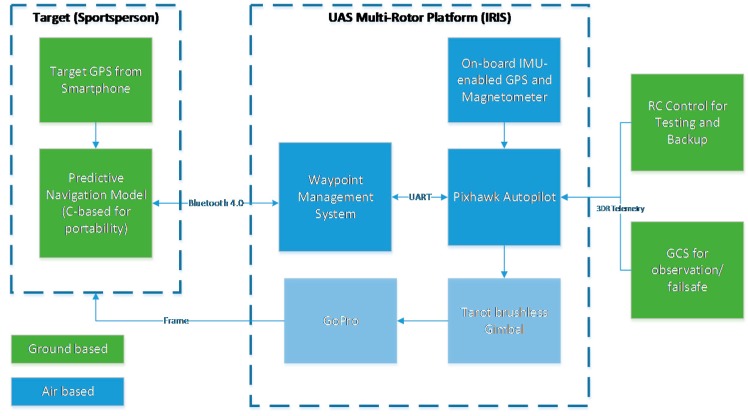
System architecture for the low-cost UAS.

**Figure 9:**
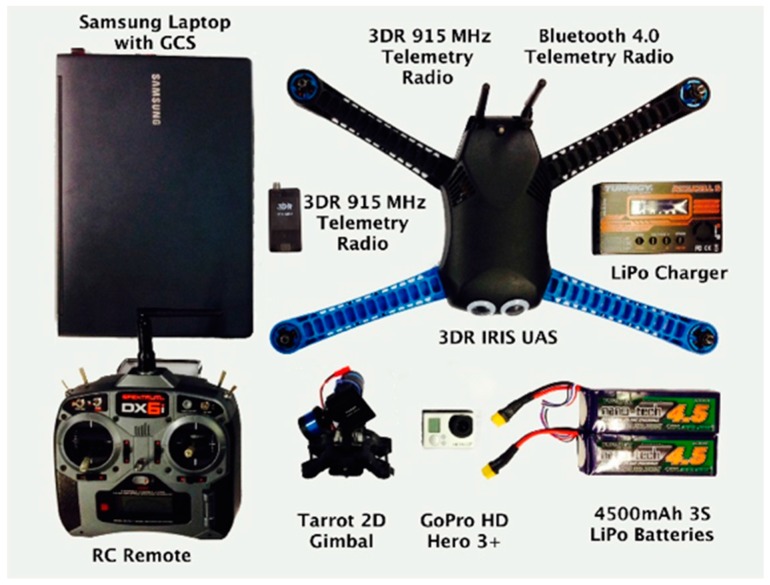
Equipment used for flight testing.

The system architecture is shown in Figure 8, and the flight test equipment is shown in Figure 9.

A commercial off-the-shelf (COTS) multirotor, the 3DR IRIS [32], was selected for testing purposes. The platform, as well as the Pixhawk flight controller were chosen for their robustness and adaptability.

A GoPro camera and a Tarot 2D gimbal were mechanically attached to the external shell of the multirotor to enable filming. To ensure that the application was able to communicate with the UAS, Bluetooth capabilities of the iPhone were utilised for micro air vehicle communication (MAVLINK) protocol implementation. A Bluetooth 4.0-based telemetry radio was developed and connected to the Pixhawk flight controller to complete this communication system. It should be noted that for testing and failsafe purposes, a multi-telemetry approach was maintained; Bluetooth was the primary data link technology, and a remote control (RC) and laptop-based GCS connected over 915 MHz were used for redundancy purposes.

Testing was conducted at an Australian Research Centre for Aerospace Automation (ARCAA) flight test facility located at Christmas Creek, Beaudesert, Queensland, Australia.

**Figure 10:**
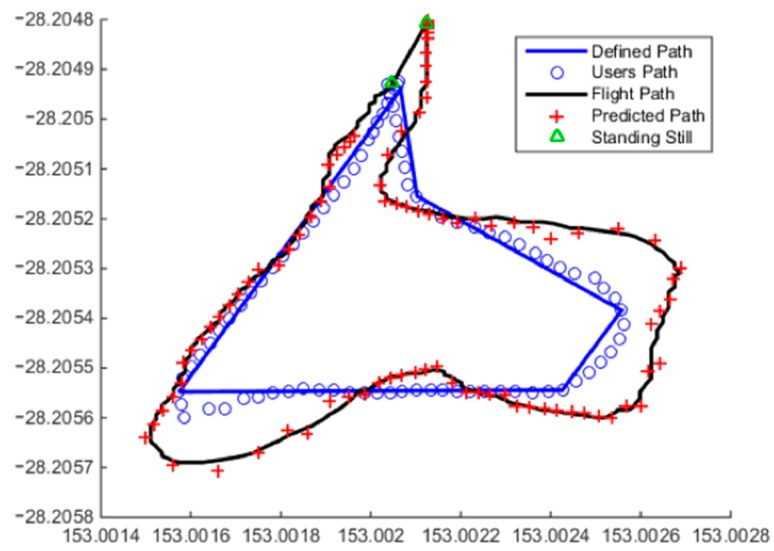
Resultant flight path, predicted waypoints, actual user path (noisy data) and the defined path demonstrating the capabilities of the system for states ${\text{Ω}}_{0}$, ${\text{Ω}}_{1}$ and ${\text{Ω}}_{2}$.

The prediction algorithm was tested over a small jogging track of approximately 270 m and 1.5 min. Figure 10 shows an example result. The solid blue line represents the path marked out for testing. The blue circles are the route taken by the user as seen from the iPhone. The red crosses are the predicted path by the algorithm output to the UAS, and finally, the black line is the path taken by the UAS.

In order to examine how well the algorithm and UAS performed, a number of criteria were assessed. The average cross-track of the user path and the UAS’ flight path was found to be 1.9 m. The mean distance the UAS was in front of the user was found to be +2.9 m. The standing still model was also tested finding no false positives, and correctly identifying all standing still cases summarises the distribution of state-to-state transitions recorded for this particular jogging track as shown in Table 2. The ${\text{Ω}}_{0}$ outputs accounted for 12% of the recorded data. Another 53% of the points were predicted with the algorithm using a previously-determined ${\text{Ω}}_{1}$ equation, *i.e.*, the UAS only moved further away from the user in the predetermined direction as per the linear HMM state definition. This is recorded as continued linear movement ${\text{Ω}}_{1}{\text{Ω}}_{1}$ and defined where a ${\text{Ω}}_{1}$ regression is reused for the next algorithm iteration. Alternatively, linear-to-adjusted linear movement is the transition from one state of linear movement ${\text{Ω}}_{1}$ to another linear movement state, meaning that a change in direction or a correction to a prior regression has been recorded. This accounted for 4% of transitions. In total, ${\text{Ω}}_{1}$ accounts for 57% of the recorded data. Non-linear movement accounts for the remaining 31%.

**Table 2:** States encountered during flight testing.

$\Omega_{0}$	Stationary	12%
$\Omega_{1}$	Linear (including continued linear and linear to adjusted-linear transitions)	57%
$\Omega_{2}$	Non-linear	31%

## 5. Conclusions

This paper presented an approach to employing a dynamic predictive waypoint navigation model to be utilised in tasks where it is desired to have a UAS ahead of a target. This system applies an HMM approach combined with assumptions regarding the continued characteristic behaviour of a target.

The accuracy of this particular prediction model could be increased with higher precision GPS receivers, including differential GPS technologies, additional modules, like IMUs, or downward-facing cameras for more information about real-time movement characteristics, or the implementation of sensor fusion by considering input measurement data from multiple independent sensors and fuzzy logic for adaptive Kalman filtering to improve localization accuracy [33]. All of these advancements need to be weighed against the cost and ease of use of the system.

Another limitation of this work is that the algorithm was developed to handle input locations as GPS positional coordinates; thus the adaptation of this project to an indoor environment, for example, would prove difficult, since deriving accurate GPS coordinates in this situation would be costly. Another major limitation applies to the UAS utilised in this paper, as there are physical constraints on the maximum airspeed and agility of the UAS that would limit the maximum velocity and acceleration that the subject would be permitted to exhibit before the UAS can no longer maintain a front-on position.

In addition to higher speed subject motion potentially exceeding physical platform capabilities, the performance of the prediction model may also be impacted. Since not only the accuracy, but also the density of input data points contributes to the input representation of test subject motion, fast-moving test subjects for same-frequency sensor systems could effectively dilute this input data and, therefore, reduce the quality of the system output.

Taking all of this into account, it can be summarised that whilst there are limitations of this system, they are predominantly balanced with keeping the system easy to use and low-cost. The algorithm has been proven to be capable of predicting the future location of a target to maintain a front-on perspective for filming purposes under the tested operating conditions. 

Future work could focus on vision-based feature tracking, sensor fusion, implementing more efficient state identification algorithms, as well as behaviour classification and recognition.

A video overview of the project is available at [34].
